# Current Use of EEN in Pre-Operative Optimisation in Crohn’s Disease

**DOI:** 10.3390/nu13124389

**Published:** 2021-12-08

**Authors:** Sharafaath Shariff, Gordon Moran, Caris Grimes, Rachel Margaret Cooney

**Affiliations:** 1 Queen Elizabeth Hospital, University Hospital Birmingham NHS Trust, Birmingham B15 2GW, UK; FathumaSharafaath.AzamShariff@uhb.nhs.uk (S.S.); rachel.cooney@uhb.nhs.uk (R.M.C.); 2 Room D1406 West Block: Queen’s Medical Centre, University of Nottingham, Queen’s Medical Centre, Nottingham NG7 2UH, UK; 3 Medway NHS Foundation Trust, Windmill Road, Gillingham ME7 5NY, UK; caris.grimes@nhs.net

**Keywords:** Crohn’s disease, exclusive elemental nutrition, pre-operative

## Abstract

Despite the increasing array of medications available for the treatment of Crohn’s disease and a focus on mucosal healing, approximately 35% of patients with Crohn’s disease undergo bowel surgery at some stage. The importance of nutritional optimisation before Crohn’s surgery is well-highlighted by surgical, nutritional, and gastroenterological societies with the aim of reducing complications and enhancing recovery. Surgical procedures are frequently undertaken when other treatment options have been unsuccessful, and, thus, patients may have lost weight and/or required steroids, and are therefore at higher risk of post-operative complications. EEN is used extensively in the paediatric population to induce remission, but is not routinely used in the induction of remission of adult Crohn’s disease or in pre-operative optimisation. Large prospective studies regarding the role of pre-operative EEN are lacking. In this review, we evaluate the current literature on the use of EEN in pre-operative settings and its impact on patient outcomes.

## 1. Introduction

Crohn’s disease (CD) is a chronic inflammatory disease of the gastrointestinal tract, with increasing incidence worldwide [1,2]. Although incidence is stabilising in western countries, prevalence continues to rise. In the U.K., it is predicted that the prevalence of inflammatory bowel disease (IBD) will be 1% by 2030 [3,4,5].

Medications such as oral immunosuppressants and biologics are the mainstay of the treatment of CD [6]. However, surgery continues to have a role in disease management. The main indications for surgery are stricturing disease, penetrating complications, and medication-refractory inflammatory disease [7].

The European Crohn’s and Colitis Organisation (ECCO) and the European Society of Coloproctology (ESCP) consensus guidelines suggest surgery should be considered at an early stage for those with penetrating or fistulising disease and those with localised ileocaecal disease and obstructive symptoms but no significant active inflammation [8]. The objective of surgery is to provide symptomatic relief and preserve as much bowel function as possible. There is a suggestion that the current, more aggressive, treat-to-target approach reduces surgical rates [9,10,11,12]. Despite these recent decreases, 23–47% of patients still require surgery at some stage in their disease course [13,14,15]. Within 5 years of diagnosis, approximately 25% of patients require surgery, with rates being the highest amongst those with ileocolonic disease. Disease recurrence is common at or close to the anastomotic site, with most patients developing new endoscopic lesions within one year of surgery, and 17–33% of patients will need a second resection in the 10-year period following the first [16].

Surgery may be the preferred first-line treatment for isolated ileocaecal CD, as shown by the Lir!c study, which demonstrated that primary resection in patients with isolated ileocecal disease had similar quality of life scores one year after surgery as compared to medical treatment [17]. 

Exclusive enteral nutrition is the term used when a patient replaces their habitual diet with an exclusive liquid diet for a defined period [18,19]. In clinical practice, the most frequent form of exclusive enteral nutrition (EEN) used is polymeric liquid feeds, for example, Fortisip™, Ensure™, or Modulen™. Polymeric feeds are more palatable, and there is no difference in outcomes between elemental and polymeric feeds (see below).

The 2020 ECCO review of peri-operative dietary therapy in IBD recognises that EEN shows promise as a pre-operative optimisation strategy for reducing complications and improving nutritional status and acknowledges a pressing need for large prospective studies to inform clinical practice [20].

The aim of EEN in CD in the pre-operative setting is to:

(1) Improve nutritional status and thus reduce surgical complications and enhance post-operative recovery [21,22].

(2) Reduce Crohn’s-related inflammation, thus reducing pre-operative steroid use and optimising the patient for surgery.

In this article, we review the available evidence to determine whether EEN can meet these aims.

## 2. Methods

This is a narrative review of the current literature (using PubMed) on the pre-operative use of exclusive enteral nutrition in Crohn’s disease (in both adults and children). Surgery refers to bowel surgery, not including perianal fistula surgery. We gathered high-quality papers, including meta-analyses, systematic reviews, randomised controlled trials, and observational data, to summarise the pre-operative role of EEN. Papers using partial enteral nutrition were also included. This review focuses only on the pre-operative use of EEN, as the role of EEN in the management of Crohn’s disease in general was already extensively reviewed elsewhere. The role of diet in the management of Crohn’s disease outside the peri-operative setting is reviewed elsewhere in this special edition. The role of parenteral nutrition or other nutritional interventions is not discussed.

We also include some practical tips about instituting EEN in your practice.

## 3. Timing of Surgery in Crohn’s Disease

Steroid dependency can occur in patients with a longstanding diagnosis of Crohn’s disease [23,24]. The most feared post-operative complications in Crohn’s disease surgery are intra-abdominal septic complications, such as anastomotic leakage, intra-abdominal abscess, and enteral fistulae [25]. Most of these patients will need drainage or reoperation and may require stoma formation [7,26,27]. 

There is a window of optimal timing for surgery in Crohn’s disease, i.e., the sweet spot. Ideally, the patient is in remission, off steroids, and well-nourished. It has been argued that the timing of Crohn’s disease surgery can be even more vital than the timing of cancer surgery; operate too soon, when the patient is on steroids, just post anti-TNF treatment, and malnourished, the risk of post-operative complications will be high. Defer surgery too long, and the patient flares again, needs steroids again, and becomes malnourished; hence, the risks rise again.

The role of the multidisciplinary team (MDT) is key in achieving optimal timing for surgery.

Other key factors in pre-operative optimisation should not be overlooked, particularly the treatment of any sepsis with radiological intervention and antibiotic therapy, and attention to venous thromboembolism risk management. In addition, this time can be used to implement smoking cessation measures. See Table 1.

### 3.1. Steroid Dependency

Although steroids have no role in the maintenance of remission in Crohn’s disease, one in four CD patients in the U.K. have prolonged exposure for greater than six months within the first 5 years of diagnosis [35]. Similar findings are also true in Europe, Canada, and the USA [36,37]. 

A meta-analysis showed steroid use to be a risk factor for intra-abdominal septic complications (odds ratio (OR) 1.99; 95% confidence interval (CI): 1.54–2.57) [28]. Low albumin and pre-operative intra-abdominal abscess formation were additional risk factors identified in this meta-analysis. Steroids delay wound healing and increase the risk of superficial and deep surgical site infections, pneumonia, myocardial infarction, renal insufficiency, and prolonged intubation [25,38,39]. Chronic steroid use significantly increases a patient’s risk of having a hospital stay longer than 30 days by 19% [40]. The risk of readmission within 30 days is increased by 58%, the risk of reoperation by 21%, and the risk of death by 32% [40].

Beaupel et al. conducted a prospective non-randomised study on patients undergoing elective surgery for ileocolonic CD [29]. Patients with obstructive symptoms, steroid treatment, pre-operative weight loss of >10%, and/or perforating CD were deemed high risk and given EEN via nasogastric tube at least two weeks prior to surgery. They used a polymeric feed enriched with transforming growth factor-beta 2 (ANS-TGF-β2) (Modulen™). Pre-operative full-dose EEN was feasible in 34/35 high-risk patients. The discontinuation of steroids pre-operatively was achieved in 10/16 patients (62.5%).

A high stoma rate in Crohn’s disease was cited in older studies with an up to 40% rate of temporary stoma formation and a 14% rate of permanent stomas at 20 years [41]. Surgeons use a stage procedure with temporary stomas if there is concern that an anastomosis will leak. The presence of risks factors, such as patients with poor nutritional status, intra-abdominal abscess, pre-operative steroid use for more than three months, and recurrent clinical episodes of Crohn’s disease, is an indication for a staged procedure [8,42,43]. EEN was shown to reduce these risk factors and thus reduce the risk of stoma formation at the time of operation. One retrospective Chinese study, which included 114 patients, found fewer stomas (22.7% vs. 40.9%) among those who received pre-operative nutritional optimisation for an average of 22 days (84.2 % received EEN, 10.5% a combination of EEN/PN, and 5.3% PN).

### 3.2. Active Disease and Intra-Abdominal Sepsis

In adults, EEN can be effective at inducing remission even in the presence of complications such as fistula/abdominal abscess formation [43,44,45,46,47].

A prospective non-randomised study in China used 12 weeks of EEN for patients with active CD with complications [34]. After 12 weeks of EEN, the Crohn’s disease activity index significantly decreased (223.43 ± 65.5 vs. 106.77 ± 42.73, *p*  ≤  0.001), and 80.5% of patients achieved full clinical remission. Fistula closure after EEN was observed in 75% of patients with enterocutaneous fistula. In patients with stenosis, 20% had no response to EEN and were transferred for surgery. Partial remission and full remission were observed in 20% and 60% of patients after 12 weeks of EEN, respectively. Intra-abdominal abscesses resolved in 76% of patients. These results are supported by the U.K. study by Heerasing et al., who found that 25% of patients were able to avoid surgery after 6 weeks of EEN. They also showed a lower incidence of post-operative abscess formation and anastomotic leakage, as well as a shorter length of stay [30]. 

A large Chinese review of 708 surgeries in a single centre assessed the impact of EEN on perioperative outcomes in four groups of CD patients: patients not exposed to immunosuppressive agents (including steroids/biologics and thiopurines) in the previous 8 weeks before surgery (group 1); patients who received immunosuppressive medications without a pre-operative drug-free interval (group 2); patients who had a pre-operative immunosuppressant-free interval (group 3); patients treated with adding 4 weeks of EEN via NG to a pre-operative immunosuppressant-free interval regimen (group 4). The patients in group 4 were found to have better outcomes, including a lower rate of stoma creation (*p* < 0.05), and a decreased incidence of post-operative infectious complications (*p* < 0.05) compared with groups 2 and 3 [48,49]. The authors suggested that EEN maintained remission off immunosuppression and improved nutritional status.

### 3.3. Malnutrition

The Global Leadership Initiative on Malnutrition (GLIM) defines malnutrition as the presence of one phenotypic criterion (weight loss, low body mass index, and reduced muscle mass) and one aetiologic criterion (reduced food intake and inflammation or disease burden) [50,51]. 

The ECCO–ESCP Consensus on Surgery for Crohn’s Disease in 2018 states that malnutrition is a significant risk factor for post-operative complications [8]. Optimisation of nutritional status is recommended before surgery via enteral or parenteral routes. It acknowledges that the evidence for using EEN to induce remission is weak. If surgery is required in malnourished patients, a staged procedure (i.e., temporary stoma formation) is advised.

One prospective study and three retrospective studies reported weight loss >10% in the six months prior to surgery in 23–54% of patients [29,42,52]. Weight loss can be multifactorial, including anorexia due to disease activity, food fear/avoidance, systemic inflammation (which alters protein synthesis and increases protein catabolism), as well as dietary restrictions due to stricturing disease [53].

Systematic reviews have confirmed the benefit of pre-operative optimisation in GI surgery in general, as well as in Crohn’s disease, but most did not focus on EEN, just nutritional optimisation through supplements [25,54,55].

Gordon-Dixon et al. recently conducted a systematic review of the use of EEN in the pre-operative optimisation of adult patients with CD undergoing intestinal resection [56]. The seven retrospective studies included in the final analysis support a trend towards improving nutritional and inflammatory markers, as well as reduced infectious complications and stoma formation, but the quality of the studies was either medium or poor, and only one study was powered to demonstrate significance (Li et al., 2015) [49]. Wang et al. demonstrated a significant increase in BMI, haemoglobin, and albumin, and a decrease in C-reactive protein (*p* < 0.01 for each). Ge et al. demonstrated a significant increase in albumin (*p* = 0.015), a reduction in CRP (*p* = 0.032), and an increase in the prognostic nutrition index (PNI) (*p* = 0.002), which is calculated using albumin and the total lymphocyte count [57]. PNI was shown to predict short-term post-operative outcomes in Crohn’s disease [57,58]. 

The retrospective study of 24 patients in the U.K. by Gordon-Dixon et al. published recently also showed no significant increase in BMI, but did show a significant increase in albumin and reduction in CRP [59]. All but three patients avoided a stoma at the time of the operation. There were three infectious complications—one anastomotic leak and two wound infections.

In two retrospective U.K. studies (Heerasing et al.; Gordon-Dixon et al.), EEN had no effect on pre-operative weight, unlike in older studies (albeit not in the pre-operative setting) [18,30,59,60]. A longer duration of therapy, e.g., three months, may be required for significant changes in weight.

## 4. Could EEN Reduce the Risk of Post-Operative Crohn’s Disease Recurrence?

The microbiome supports intestinal homeostasis and the immune function, and it is widely accepted that disruptions in host–microbiome interactions are the driving force behind tissue-damaging inflammation in CD [61]. Although the exact mechanism of action of EEN is not known, the current hypothesis suggests it works through the exclusion of dietary components that interact with inflammatory components of the gut microbiome [62,63]. 

Many research studies found that compared to CD patients, healthy subjects had high biodiversity, dominated by Firmicutes, Bacteroidetes, and Proteobacteria phyla [64]. Biodiversity is lower in CD patients at the time of surgery, but is increased after surgery, whilst still different from healthy subjects [65]. Bacterial dysbiosis and a low abundance of Faecalibacterium *prausnitzii*, in both resected and post-operative ileal mucosa, have been associated with an increased risk of endoscopic recurrence [66,67,68]. One study looked at the post-operative Crohn’s recurrence rates after at least 4 weeks of EEN [57]. At 6 months after laparoscopic surgery, the rate of endoscopic recurrence was significantly lower in the EEN group ((11.9% vs. 28.4%, *p* = 0.044). At 12 months, the difference, however, was not significant (26.2% vs. 37.3%, *p* = 0.059). The rates of clinical recurrence at 12 months were four (8.9%) patients in the EEN group and nine (12.0%) patients in the non-EEN group (*p* = 0.820) [57]. Potentially, the continuation of EEN or PEN post-operatively could result in lower long-term recurrence rates. Cohort studies found that nocturnal EN in the post-operative period may reduce clinical and endoscopic recurrence among adult patients with CD [69,70]. Assessing the changes in the microbiome during EEN could reveal which microbes and microbial metabolites play a role in the aetiology of CD.

## 5. EEN Use in Practice

Interest in therapeutic diets has grown in recent years, and questions regarding diet are common in IBD clinical care. Dietary advice is part of the personalised care plans advocated by national bodies [6].

EEN is not routinely used to induce or maintain remission in adult populations. Anecdotally, some centres use EEN pre-operatively in selected patients, namely those who are malnourished and at high risk of surgical complications, to improve nutrition, wean them off steroids, and even allow a period of smoking cessation prior to operation. It may be that in adults, EEN is most effective in this setting. Routine use has not been adopted due to a combination of factors, including the lack of robust prospective studies, perceived compliance issues, patient preference, and limited access to dietetic expertise within an IBD multidisciplinary team [71].

Patients may be more willing to comply with EEN if this could reduce the risk of post-operative complications, including stoma formation, and allow a shorter hospital stay and a faster return to work. Stoma formation is something many patients are keen to avoid, and this may be a factor in patient compliance [72,73].

To help address this, an Australia and New Zealand working group developed a care pathway for EEN use in adults with CD [74].

Many questions arise when considering EEN. A common question is whether all daily calorie intake must be from formula feeds. Recent studies showed partial enteral nutrition together with a specific CD exclusion diet can induce remission in children and adults [75,76,77]. This may help compliance, but there are currently no studies of this in the post-operative setting.

Many of the studies in China used an NG tube to deliver feed, but the efficacy of EEN in CD is independent of the route of administration, and in our experience, patients most commonly opt for oral rather than the NG route [45].

The type of formula feed used varies across studies, but if we extrapolate from the paediatric population, one formula is not superior to another [78]. This means that an adult patient who does not tolerate one form of formula feed can be offered an alternative until a feed is found that the patient can tolerate. Of note, in the study by Gordon-Dixon et al., Modulen™ IBD was used as first-line treatment, but if this was not tolerated, Ensure™ was offered, with a liquid diet as third-line treatment. Of the 24 patients in this study, 17 tolerated Modulen™, 5 Ensure™, and 2 a standard liquidised diet.

## 6. Conclusions

In conclusion, retrospective and single-centre prospective studies of adult patients suggest that EEN may pre-operatively optimise the patient, thus reducing the risk of surgical complications. However, large prospective studies are urgently required to confirm its effectiveness, define its use, and define the mechanism involved. The role of PEN vs. EEN in this period also deserves further research, as well as whether continuing PEN/EEN post-operatively is beneficial. There is growing interest in diet as therapy and greater understanding of the microbiome, which may in time lead to the tailoring of both dietary and medical intervention in a truly personalised care plan.

## Figures and Tables

**Table 1:** Key risk factors for complications.

Risk Factor	Evidence for Role of EEN—References
Steroid dependency [28]	Yes [29,30,31]
Active disease [32]	Yes [30]
Sepsis [28]	Drain if possible. EEN may assist in management alongside antibiotics [30,33,34]
Impaired nutritional status [32]	May help
